# A New Military Hospital in Paris

**Published:** 1860-07

**Authors:** 


*A New Military Hospital in Paris.*—The Minister of War has lately decided that a military hospital shall be established in the northern part of Paris. The principal hospital of the kind, called Val de Grace, is situated, as is well known, on the left or southern side of the Seine. It has been proposed to use the Hospital for Incurables for the purpose, and to transfer the patients of the latter institution into a new building to be erected outside the fortifications. Another part of the project is to place all the hospitals in which very chronic or incurable cases are received beyond the fortified line. What is to become of the patients in the event of a seige?

